# Evaluation of an Adverse Childhood Experiences Screening Program for Pregnant Patients in Rural Missouri

**DOI:** 10.1007/s40653-026-00890-7

**Published:** 2026-05-02

**Authors:** Aiman Fatima, Ellen M. Chiocca, Jennifer Blacksmith, Melodie D. Stocks

**Affiliations:** 1 Department of Nursing, Temple University Barnett College of Public Health, Paley Hall, 1210 W. Berks Street, Philadelphia, PA 19122 USA; 2 Sinclair School of Nursing, University of Missouri, Columbia, MO USA; 3 Behavioral Health, Northeast Missouri Health Council, Kirksville, MO USA; 4 OB/Gyn Specialty Group, Northeast Missouri Health Council, Kirksville, MO USA

**Keywords:** Adverse childhood experiences, Pregnant patients, Screening, Referrals, Trauma informed care

## Abstract

Adverse Childhood Experiences (ACEs) are defined as exposure to traumatic conditions from birth until age 18 years. Exposure to ACEs is associated with poor health outcomes and increased risks of chronic conditions such as obesity, heart disease, diabetes, stroke, and cancer. Pregnant women who have experienced ACEs have increased rates of depression, anxiety, post-traumatic stress symptoms, substance abuse and suicidality. The high prevalence of ACEs justifies screening, early support and interventions. This Quality Improvement Project was an evaluation of an existing ACEs screening program initiated in 2019 at an Obstetrics and Gynecology clinic in Northeast Missouri. It was aimed at screening ACEs in pregnant patients at their prenatal visit, so that timely referrals and resources can be provided to patients with high ACEs scores. The effectiveness of this program was evaluated by screening charts of all the eligible patients January 2021 to March 2021 (Timepoint 1, T1) and January 2022 to March 2022 (Timepoint 2, T2). Of the 144 patients, 50 participants (34.7%) had an ACE score of three or above. There was no statistically significant difference in the ACE scores between T1 and T2 (*p* = .21). Although not statistically significant, there was a small to moderate increase in Community Health Worker (CHW) referrals for ACE scores of four or higher. There was a moderate, statistically significant increase in the number of patients who kept their appointment from T1 to T2, c^2^ (4) = 9.74, *p* = .05, ϕ = 0.26). There was a 300% increase in referrals provided to pregnant patients with ACE scores of four or higher from T1 to T2. These changes could stem from the fact that every three months, providers are now prompted about ACEs, screening, documentation, and Trauma Informed Care (TIC). This has ensured that providers have ACEs and TIC awareness thereby facilitating timely referrals of patients to appropriate resources.

## Introduction

Adverse childhood experiences (ACEs) are defined as exposure to traumatic events from birth until age 18 years (Felitti, et al., 1998). These conditions include physical or emotional abuse/neglect, sexual abuse, and household dysfunction, and have been shown to be associated with an increased lifetime risk of chronic diseases such as obesity, heart disease, diabetes, stroke, and cancer (Felitti, et al., 1998; Sherfinski et al., 2021). ACEs are very common in the United States. Two-thirds of adults have at least one type of ACEs; 25% of Americans report three ACEs while 16% have experienced at least four ACEs (Miller et al., 2021; Young-Wolff et al., 2019). Moreover, the impact of maternal ACEs during pregnancy has been associated with increased depression, anxiety, post-traumatic stress symptoms, substance abuse and suicidality (Young-Wolff et al., 2019). ACEs may also increase the likelihood of an unintended, unwanted, or a mistimed (occurring earlier than desired) pregnancy. Both ACEs and unintended pregnancies are linked to higher risk for maternal mental health problems during pregnancy and to negative pregnancy outcomes (Testa et al., 2021; Young-Wolff et al., 2019). ACEs in a pregnant mother can also lead to poor child health and abnormal child development until at least four years of age (Iyengar et al., 2019; Osofsky et al., 2021; Young-Wolff et al., 2019). The high prevalence of ACEs justifies screening and early support and interventions in pregnant women (Esden, 2018; Rariden et al., 2021).

## Background and Significance

Because maternal stress and inadequate social support are well-established risk factors for adverse pregnancy outcomes (Jagtap et al., 2023; Renbarger et al., 2021), more efforts to incorporate ACEs screening in primary care and prenatal settings have emerged (Luckett et al., 2025; McBain et al., 2023). The purpose of ACEs screenings in pregnant women is to promote healthy child development across life by proactively supporting safe, stable and nurturing relationships (SSNRs). The ACEs assessment also enables teaching resilience, intervening early on to promote healing the trauma and stress associated with the disruptions in the SSNRs (Bethell et al., 2017). Pregnant women who are at low risk, without associated health conditions also need to be provided with education about ACEs, toxic stress, and health coping strategies, i.e., resilience (California Department of Health Care Services (CDHCS), 2020).

This underscores the need for health care providers and staff to be educated regarding Trauma Informed Care (TIC), a resilience model that focuses on patient healing while recognizing the impact of childhood trauma on the individual (Esden, 2018; Leitch, 2017; Ranjbar & Erb, 2019). The TIC model incorporates the unique challenges and adaptations required to address ACEs. The 4Es model is a subset of the TIC model (Esden, 2018). It was initially developed to address trauma in women residing in correctional facilities. The 4Es model can be adapted for patients with ACEs in primary care and consists of four elements: *Educating* the staff about ACEs, *Empathizing* with the patient, *Explaining* the details about ACEs to the patients and finally, *Empowering* the patients by using a patient centered approach and shared decision making (Esden, 2018).

The successful implementation of the TIC model requires incorporation of the TIC pyramid. In this pyramid, screening is followed by the clinician’s self-awareness, interprofessional collaboration, understanding the health impacts of trauma and adopting a patient centered communication and care approach (Marcoux, 2021). In pregnant patients with high ACEs, resilience training has been shown to be an effective modality. This includes emotional regulation training, reframing thoughts and positive emotions through cognitive and behavioral approaches while improving their physical health through exercise, nutrition, relaxation, social support, and mindfulness (Young-Wolff et al., 2019).

## Review of Literature

A systematic literature review of descriptive studies by Rariden et al. (2021) suggests that providers often lack adequate training, time and education related to ACEs. The authors found that these barriers lead to a lack of confidence and a certain level of discomfort in discussing past trauma with a patient. A systematic review of randomized control trials or quasi experimental designs with control groups was also carried out by Loveday et al. (2022). The authors found that the baseline rate of referrals by primary care providers was very low, between two to eight%. A lack of evidence-based interventions and trauma-informed care models for prenatal setting restricts addressing positive scores. Also, providers often fear that discussion about ACEs can trigger patient re-traumatization. A mixed methods study by Flanagan et al. (2018) indicated that three in 104 patients who had ACEs score of more than or equal to one, reported limited time constraints, lack of provider empathy and available resources. The mixed methods study by Gillspie and Folger (2017) found that 61% of the providers had insufficient training, lack of TIC knowledge and resources to address patients with high scores. In a retrospective study by van Rossel et al. (2021), a total of 338 ethnically diverse patients self-reported their ACEs scores in their second prenatal visit. Due to a stigma associated with mental health issues or a discomfort in discussing sensitive issues, only 32% of the pregnant women reported ACEs scores of one or above while 14.4% declined the screening.

Nguyen et al. (2019) conducted an observational study which included women 18 years or older receiving prenatal care. Study results revealed that providing privacy during screening helped to ensure client participation. Of the subjects, 13.3% of the participants refused screening if it was conducted in the waiting room whereas less than 4% declined it in the examination room. An observational study by Kalmakis et al. (2018) found that 80% of the interviews lasted ten minutes or less when the health care providers received a two-hour TIC training. The ACEs scores and time to screen were found to be positively correlated. A mixed methods study by Kia-Keating et al. (2019) included 164 parents and indicated a high level of acceptability of screening with 92% of parents receiving ACEs screening and 77% of eligible parents consenting to avail available resources. The screening acceptability was higher when it was provided by a trusted provider and there was a plan to address the high scores.

A quality improvement study by Watson et al. (2022) examined the perspectives of 119 pregnant women on ACEs and resilience screening. The majority (94.0%) of the women in this study believed that it was important to have conversations about resilience during the screening. Women with high ACEs scores, and women with low resilience preferred longer discussion times about ACEs. Most of the participants believed that provider empathy could make the ACEs conversations more effective. Nearly half used the handout about resources provided to them. During a quality improvement project at a pediatric primary care clinic conducted by Bryant and VanGraafieland (2020), 59 providers received ACEs education and a symptoms checklist to determine the need for referrals. This improved the ACEs knowledge of the providers and awareness about available resources.

## Background/Aim of Project

This Quality Improvement (QI) project is an evaluation of an existing ACEs screening program which was implemented at a prenatal Obstetrics and Gynecology clinic in Northeast Missouri in 2019. The purpose of this QI project was to examine how this program, which screens pregnant patients to identify ACE scores of three or higher, affects the rates of patient referrals/provision of relevant social service resources.

## Methods

### Setting

The setting in which this QI project took place is an Obstetrics and Gynecology clinic in Northeast Missouri and is a not-for- profit corporation with a rural designation meeting the healthcare needs across nine counties in the area. The clinic has an Obstetrics and Gynecology Group that at the time of this study comprised of two Doctors of Osteopathic Medicine (DO) and a women’s health nurse practitioner. The clinic serves a diverse, rural population including low-income, uninsured, and Medicaid/Medicare patients.

### Intervention and Procedure

To initiate this QI project, the project lead and clinic manager met to discuss the goals of the project. A formal presentation on best practices regarding screening for ACEs and TIC was prepared and then presented to clinic staff and providers (see the timeline below under “Data Collection Procedures”). In 2022, a report was given to the providers indicating which patients did not have ACEs scores documented. Subsequently, ‘patient needs ACEs’ reminder was connected to the patient’s name in the inbox helping nurses to remember to ask patients for their ACEs scores. It has become a required EMR documentation for the providers. Every patient is informed about the ACEs screening tool in the examination room by the nursing staff. The patient also has the option to refuse the screening. The ACEs screening questionnaire is provided as a laminated sheet and is attached to the clipboard. The patient is then required to mentally checkmark the ACEs categories that the patient might have experienced before the 18th birthday. The ACEs score is the total of the checked responses in the tool. The patient can provide the total score to the nursing staff without mentioning the specific ACEs categories. This score is then documented in the EMR by the provider. The providers are required to check this documentation of the ACEs score for the patient, reeducate the patient about the need to do the ACEs scores and ascertain if a referral is needed based on the scores. If the ACEs score is intermediate or high the provider discusses the need for referral and the available resources with the patient. A referral to a Community Health Worker (CHW) is usually made by the provider when the ACEs score is three or above. At the clinic, the ACEs scores of the patients automatically transfer from the Electronic Medical Record (EMR), then to Power Bi.

### Inclusion/Exclusion Criteria

The inclusion criteria included ‘obstetrics’ codes such as from less than 8 weeks gestation of pregnancy to greater than 42 weeks gestation. Also, ICD-10 codes such as encounter for supervision of normal pregnancy first trimester (Z34.01), encounter for supervision of normal pregnancy second trimester (Z34.02), encounter for supervision of normal pregnancy third trimester (Z34.03) and encounter of pregnancy of unspecified trimester (Z34.00) were used. Patients also had additional ICD 10 codes in addition to the ones specified such as ‘abnormal glucose complicating pregnancy’ O99.810 and ‘smoking (tobacco) complicating pregnancy’ O99.330. Exclusion criteria included patients who did not meet the ‘obstetrics’ codes or the encounter type criteria. Demographic information includes age, race, marital status and education level.

### Data Collection Procedures

For the project’s medical record review, a retrospective chart review was done in Power Bi. The organization’s Director of Clinical Informatics generated reports in Power Bi which included all the patients meeting the inclusion criteria for the specific time frames relevant to this project. Hence, there were no missing or incomplete charts. For data collection, the primary investigator then utilized this data of the participants from the Power Bi.

The effectiveness of the ACEs screening program was evaluated by screening the lists of all the eligible patients in Power Bi from January 2021 to March 2021 (T1) and January 2022 to March 2022 (T2). The time points of 2021 and 2022 were chosen based on the implementation of the ACEs screening tool at the clinic and the subsequent meetings with the staff. The tool itself was introduced in 2019. An initial meeting with all the staff regarding ACEs was held in 2018. The clinic director delivered a PowerPoint presentation on TIC and best practices of ACEs in 2020. This information was presented to the staff and the providers at two separate meetings that year. In 2022, the clinic director had another meeting regarding ACEs with the providers.

### Standardized Tools

The ACEs screening questionnaire used for this project has been adapted from the original work of Kaiser Permanente and CDHCS (CDHCS, 2026; Felitti et al., 1998) (Appendix, Fig. 4). The tool has high reliability and validity with the initial testing done in the context of the original study (Bethell et al., 2017). Several other ACEs assessment tools have been identified (Bethell et al., 2017). For example, the National Survey of Children’s Health (NSCH) ACEs, Behavioral Risk Factor Surveillance Survey (BRFSS), World Health Organization (WHO) ACE International Questionnaire and the National Survey of Child and Adolescent Wellbeing (NSCAW). The NSCH, The BRFSS and the NSCAW are based on the original CDC/Kaiser study. The NSCH ACEs is an adult screening tool and has been shown to have strong internal validity. The BRFSS, an adult screening tool, includes concurrent screening of other health issues as well. The WHO ACE International Questionnaire includes questions to incorporate cultural applicability. It is currently undergoing reliability and testing studies. NSCAW includes adults discussing their children. It has questions from already validated screening tools such as the childhood trauma scale (Bethell et al., 2017). The American College of Obstetricians and Gynecologists (ACOG) recommend universal screening of present and past experiences of trauma but does not indicate the best way of screening (ACOG, 2021).

### ACE Scores

Based on the ACEs screening tool for adults, a score of zero is associated with no risk, a score of one to three indicates intermediate risk, while a score of four and above is considered a high-risk score (CDHCS, 2026).

### Operational Definition of Variables

The documentation of the ACEs scores was defined as the presence of a completed ACEs screening score recorded in the EMR of the patient. Gravida was defined as the total number of documented pregnancies in the EMR including the current pregnancy and pregnancies ending in live birth, still birth, miscarriage or abortion. Term pregnancy was defined as a documented delivery occurring at more than equal to 37 completed weeks of gestation. Preterm pregnancy was defined as a documented delivery occurring between 20 and 36 weeks of gestation. Live births were defined as deliveries in which the infant showed signs of life after birth as documented in the obstetric history of the patient in the EMR. The CHW referral was defined as the documentation of a referral by the provider in the EMR when the ACEs score was three or above.

### Ethical Considerations

The Institutional Review Board at the University of Missouri, Columbia, MO determined that this study does not constitute human subjects research according to the Department of Health and Human Services Regulatory Definitions. As such, no IRB review/requirements were needed. Since this is a quality improvement project and entails an evaluation of an existing program, there is minimal patient risk.

## Measures

### Analysis

Using a confidence interval of 95%, a 5% margin of error, and a population size of 88, a minimum of 72 charts were required for review at each timepoint (Raosoft, 2004). To ensure an unbiased representation of the sample, the 72 charts were randomly selected for review. Different charts were reviewed at each time point. The primary outcome variable was documentation of the ACEs scores. Secondary outcome variables included: documentation of the pregnancy outcomes such as gravida, term pregnancy, pre-term pregnancy, abortions, live births, referral to community health workers, appointments kept and provision of counseling. Descriptive statistics were utilized to provide an overview of the project sample. Nominal level data was analyzed with the Chi-square Test of Independence, and the *phi* coefficient was used as an index to describe the magnitude of the effect from the intervention with values 0.10, 0.30, and 0.50 corresponding with small, moderate, and large respectively. Ratio level data was analyzed using the independent *t*-test. IBM SPSS Statistic version 24 (Chicago, IL) was used for statistical analysis. Statistical significance was defined as *p* ≤.05.

## Results

### Demographics

Demographic data was collected on all participants. There were 144 patients in the sample, 72 in T1 and 72 in T2. The predominant age group was 21–30 (64.6%, *n* = 93), followed by 31–40 (29.9%, *n* = 43), 17–20 (2.8%, *n* = 4), 41–45 (2.1%, *n* = 3), and 46–50 (0.7%, *n* = 1). The sample was primarily white (88.2%, *n* = 127), followed by unreported (6.3%, *n* = 9), Black/African American (4.9%, *n* = 7), and more than one race (0.7%, *n* = 1). The sample was mostly married (49.3%, *n* = 71), followed by single (44.4%, *n* = 64), divorced (2.8%, *n* = 4), unreported (2.1%, *n* = 3), and widow (1.4%, *n* = 2).

The predominant educational level of the sample was unreported education (52.8%, *n* = 76), followed by high school (23.6%, *n* = 34), college/community college/trade school (17.4%, *n* = 25), high school not completed (3.5%, *n* = 5), and some college (2.8%, *n* = 4). There were no statistically significant differences found between the groups in age (*p* =.20), race (*p* =.53), marital status (*p* =.52), or education (*p* =.22). The demographic data is shown in Table 1.

**Table 1:** Overall demographics

Age	21–30 y	31–40 y	17–20 y	41–45 y	46–50 y
	64.6%, *n* = 93	29.9%, *n* = 43	2.8%, *n* = 4	2.1%, *n* = 3	0.7%, *n* = 1
Race	**White**	**Unreported**	**Black/African American**	**More than one race**	
	88.2%, *n* = 127	6.3%, *n* = 9	4.9%, *n* = 7	0.7%, *n* = 1	
Marital Status	**Married**	**Single**	**Divorced**	**Unreported**	**Widow**
	49.3%, *n* = 71	44.4%, *n* = 64	2.8%, *n* = 4	2.1%, *n* = 3	1.4%, *n* = 2
Education Level	**Unreported Education**	**High School**	**College/Community College/Trade School**	**High School not completed**	**Some College**
	52.8%, *n* = 76	23.6%, *n* = 34	17.4%, *n* = 25	3.5%, *n* = 5	2.8%, *n* = 4

**Fig. 1:**
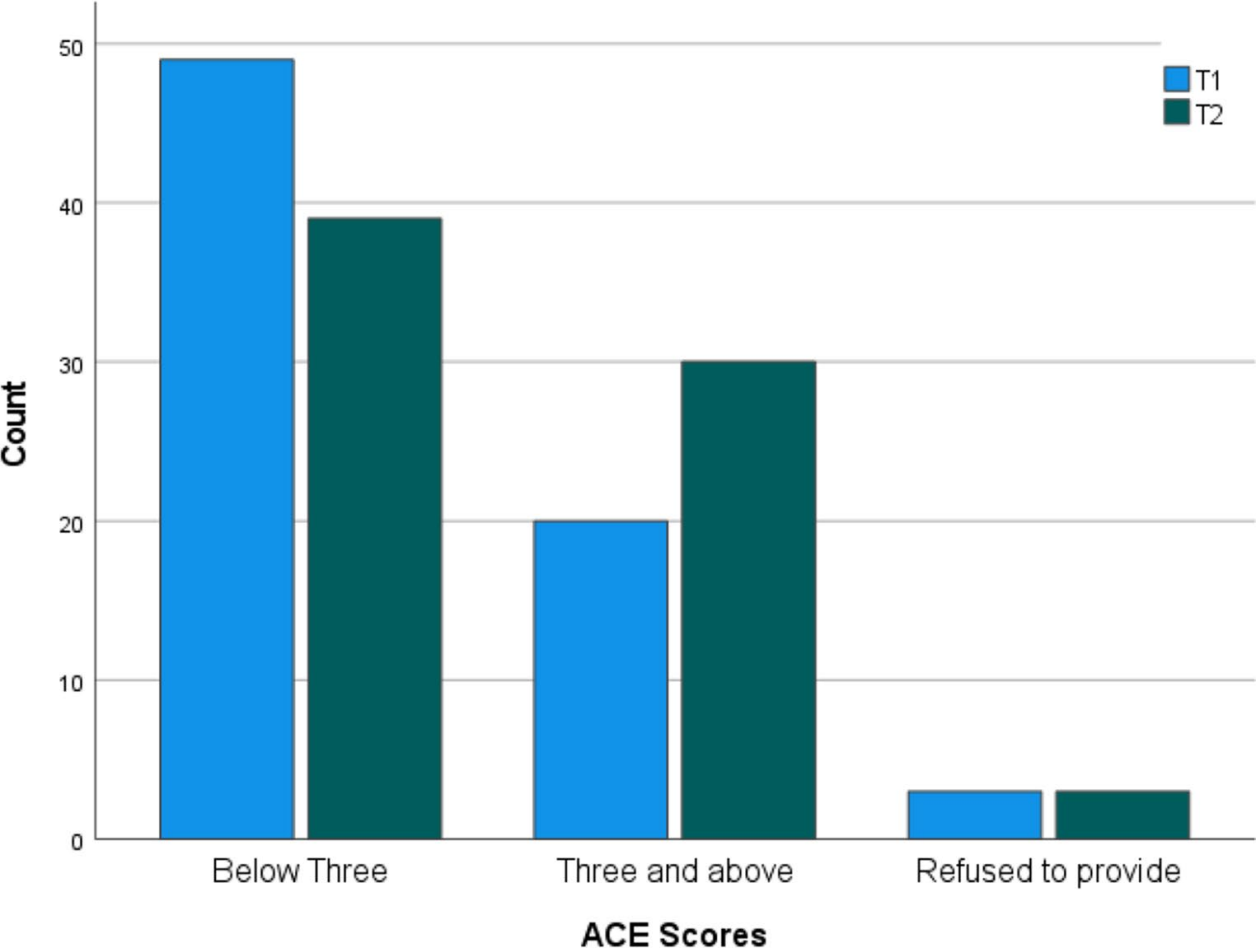
ACE Scores

Of the 144 patients, there were six who refused to provide ACE scores. Of the remaining 138, 69 patients in T1 had a mean ACE score of2.09 (SD= 2.53). Similarly, the 69 patients in T2 had a mean score of 2.53 (SD= 2.72). The difference between the two means was notstatistically significant (p=.32). The predominant ACE category was those patients who scored below three (61.1%,n= 88). There were 50(34.7%) who scored three and above (n= 50) with six who refused to provide any ACE score (4.2%). There was no statistically significantdifference in the ACE scores between T1 and T2 (p=.21). The ACE scores for T1 and T2 are shown in Fig. 1.

### ACE Scores Based on Race

Of the seven Black patients in the sample, 57.1% (*n* = 4) had an ACE score of three or above. Conversely, of the 127 white patients in the sample, only 13.4% (*n* = 17) had an ACE score of three or above.

### ACE Scores Based on Demographic Data

Of the 50 patients with an ACE score of three or above, five (10%) had an ICD 10 code of extreme poverty. Of the 50 patients with an ACE score of three or above, 70% (*n* = 35) were 21–30 years of age. Of these 35 patients, 33 (94.3%) did not have an ICD 10 code of extreme poverty and 31 (93.9%) were white.

Of the 31 white patients, 54.8% (*n* = 17) did not report an education level while 22.6% (*n* = 7) had college/community college/trade school education level. There were six (19.4%) high school graduates and one (3.2%) who had not completed high school. Of the 35 patients, one 2.9% (*n* = 1) had an unreported race and an unreported education while 2.9% (*n* = 1) was black/African American with an incomplete high school education. Of the 35 patients, 5.7% (*n* = 2) had an ICD 10 code of extreme poverty. Of the two patients, 50% (*n* = 1) was of white race with high school education while 50% (*n* = 1) was of Black/African American race with some college education.

Of the 50 patients with an ACE score of three or above, 26% (*n* = 13) were in the age group of 31–40 years. Of the 13 patients, 76.9% (*n* = 10) did not have an ICD 10 code of extreme poverty. Of the 10 patients, 50% (*n* = 5) were white with high school education, 30% (*n* = 3) were white with an unreported education, 10% (*n* = 1) were of more than one race with a college/community college/trade school education and 10% (*n* = 1) were Black/African American with an unreported education. Of the 13 patients in the age group of 31–40 years, 23.1% (*n* = 3) had an ICD 10 code of extreme poverty. Of these three patients, 66.7% (*n* = 2) were both white with unreported education and 33.3% (*n* = 1) had an unreported race and a high school education.

Of the 50 patients, 2% (*n* = 1), were in the 41–45 years of age, did not have an ICD 10 code of extreme poverty, were of Black/African American race with an unreported education. Of the 50 patients, 2% (*n* = 1) were 17–20 years of age, did not have an ICD 10 code of extreme poverty, and were white with an unreported education. The ACE scores based on this demographic data are shown in Table 2.

**Table 2:** ACE scores based on demographic data

Age					No ICD 10 code of extreme poverty	ICD 10 code of extreme poverty		
21–30 y (*n* = 35, 70%)	93.9% (*n* = 31) white				94.3% (*n* = 33)	5.7% (*n* = 2)		
	**Education Not Reported**	**College/Community College/Trade School Education**	**High School Graduates**	**No High School**		50% (*n* = 1) White race with high school education	50% (*n* = 1) Black/African American race with some college education.	
	54.8% (*n* = 17)	22.6% (*n* = 7)	19.4% (*n* = 6)	3.2% (*n* = 1)				
31–40 years (*n* = 13, 26%)	**Of the 10 patients with no ICD 10 of Extreme Poverty**				76.9% (*n* = 10)	23.1% (*n* = 3)		
	50% (*n* = 5) were white with high school education	30% (*n* = 3) were white with an unreported education	10% (*n* = 1) more than one race with a college/community, college/trade school education	10% (*n* = 1) Black/African American with unreported education.		66.7% (*n* = 2) Both white with unreported education.		33.3% (*n* = 1) Unreported race and a high school education.
17–20 years (*n* = 1, 2%)					2% (*n* = 1)			
					White race with unreported education			
41–45 years (*n* = 1, 2%)					2% (*n* = 1)			
					Black/African American race with unreported education.			

Demographic data of the 50 patients with ACEs scores of three or above

### ACE Scores and Pregnancy Outcomes

There were no statistically significant correlations between ACE scores and abortions (*r*=.12, *p* =.17), gravida (*r* = −.01, *p* =.95), preterm pregnancy (*r* =.11, *p* =.19) or live births (*r* = −.01, *p* =.26). There was a small statistically significant negative correlation between ACE scores and term pregnancy, *r* (136) = − 0.19, *p* =.03.

### Referral to Community Healthcare Worker (CHW)

Of the 144 patients, 73.6% (*n* = 106) did not need any referral, as they did not meet the criteria. Of the remaining 34 patients, 13.2% (*n* = 19) met the criteria for a referral but the referral was not provided. Of the remaining subjects, 10.4% (*n* = 15) received a referral and 2.8% (*n* = 4) received a mental health referral. Although not statistically significant, there was a small to moderate increase in CHW referrals between T1 (4.2%, *n* = 3) and T2 (16.7%, *n* = 12), *p* =.09, ϕ = 0.21.

### CHW Appointments Kept

Of the 144 patients, 126 patients either did not meet the criteria for a referral, were not provided a referral though they needed it or were provided with a referral for clinical psychology or psychiatry. Of the remaining 18 patients, 4.2% (*n* = 3) kept their CHW appointments in T1 as opposed to 16.7% (*n* = 12) in T2. There was a moderate, statistically significant increase in the number of patients who kept their appointment from T1 to T2, c^2^(4) = 9.74, *p* =.05, ϕ = 0.26).

### Counseling

**Fig. 2:**
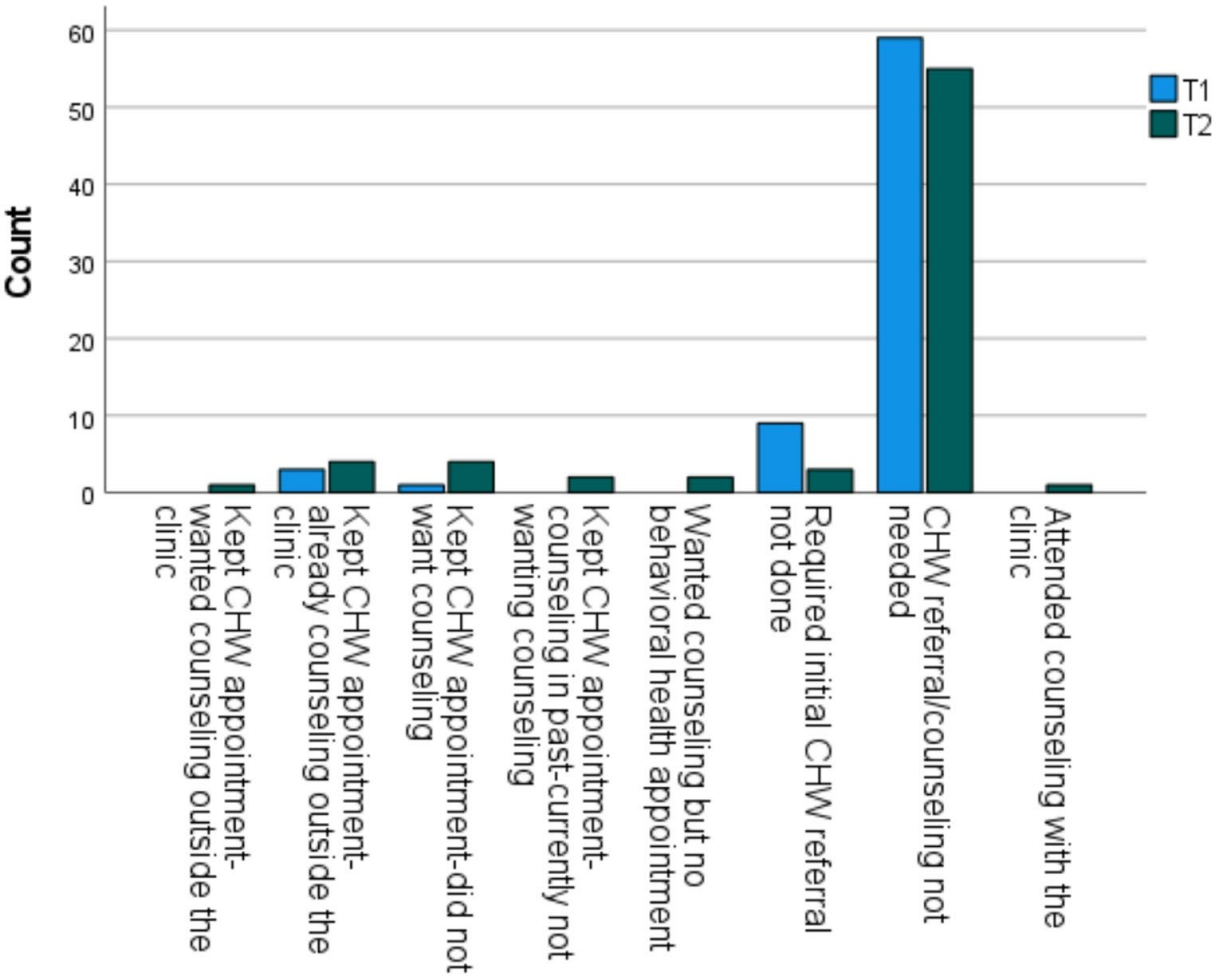
Counseling Note. Details of CHW and counseling for patients at the clinic in Northeast Missouri

**Fig. 4:**
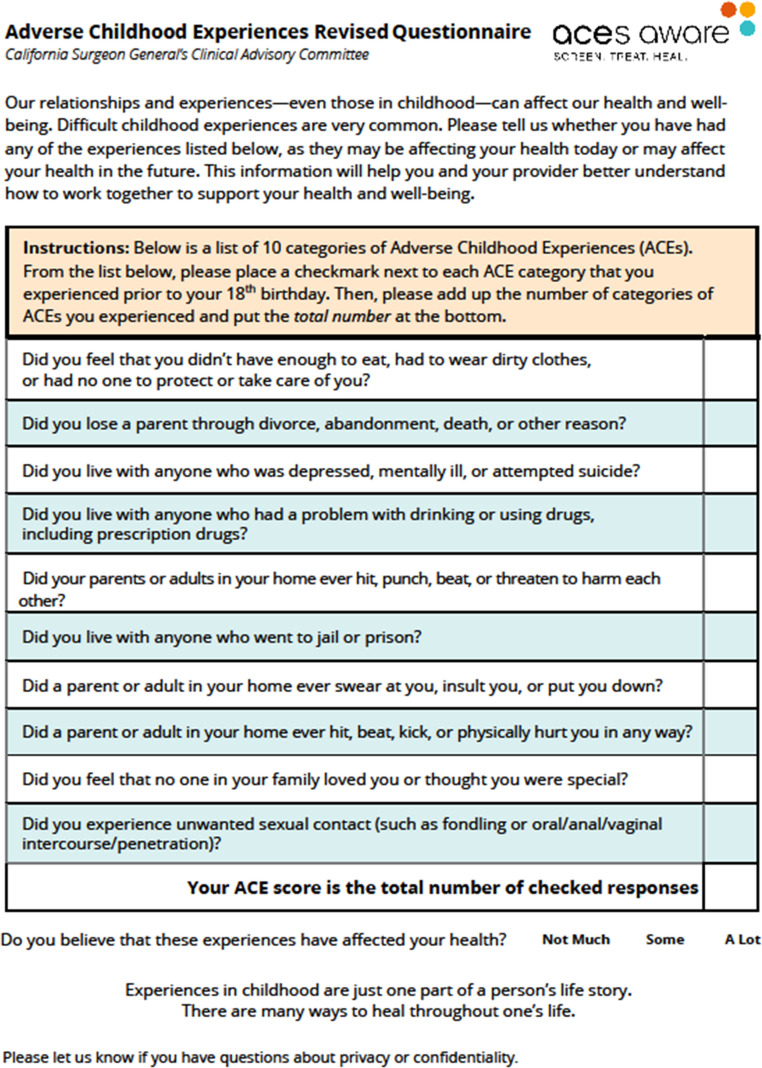
The adverse childhood experiences revised questionnaire

Of the 144 patients in the sample, 79.2% (*n* = 114) did not need counseling or a CHW referral, 8.3% (*n* = 12) needed a CHW referral but did not receive it, 4.9% (*n* = 7) kept the CHW appointment but were already receiving the counseling outside the clinic, 3.5% (*n* = 5) kept the CHW appointment but refused counseling. Further, 1.4% (*n* = 2) kept the CHW appointment, had received counseling in the past and did not want to receive counseling, 1.4% (*n* = 2) wanted counseling but did not schedule a behavioral health appointment for it, 0.7% (*n* = 1) kept CHW appointment but wanted counseling outside the clinic and 0.7% (*n* = 1) attended counseling with the clinic in Northeast Missouri. Although not statistically significant, there was a moderate decrease in the unprovided required initial CHW referrals from 12.5% (*n* = 9) in T1 to 4.2% (*n* = 3) in T2 (*p* =.14, ϕ = 0.28). The details of the CHW referrals and counseling are shown in Fig. 2.

## Discussion

The current QI project was aimed at screening ACEs in pregnant patients so that timely referrals and resources could be provided to patients with high ACEs scores. The first project goal, a 5% increase in the EMR documentation of the ACEs scores from T1 to T2 was not met since there was a 0% increase, 69 scores were documented at each time point. The second of a 3% increase in referrals provided to pregnant women with ACEs scores of three or higher from T1 to T2 was met with a 300% increase in referrals. Since the providers at the clinic have been providing referrals to patients with an ACE score of four or higher, this was incorporated in the analysis of referrals. Although not statistically significant, there was a small to moderate increase in CHW referrals for ACE scores of four or higher from 4.2% in T1 (*n* = 3) to 16.7% (*n* = 12) in T2. Although not statistically significant, there was a moderate decrease in the required initial CHW referrals which were not provided from 12.5% (*n* = 9) in T1 to 4.2% (*n* = 3) in T2. Also, there was a moderate statistically significant increase in patients who kept their appointment from T1 to T2. These changes could stem from the fact that during 2022, providers had been asked about ACEs, ACEs documentation and Trauma Informed Care (TIC) awareness. They were also encouraged to ask any questions about ACEs. This reflects a component of the 4Es model, namely educating the providers about ACEs in primary care so that they can further empathize, educate and empower the patients (Esden, 2018).

## Strengths and Limitations

This QI project was instrumental in underscoring the importance of both educating the staff about the importance of screening for ACEs and carrying out this screening on vulnerable clients. For sustainability, the clinic management is preparing to incorporate the provision of referrals for patients at an intermediate risk. There is also a plan to provide TIC training to providers once a year. The plan for future projects includes focusing on the relation of the high ACE scores with the comorbidities of the pregnant patients.

Some of the limitations of this project included unavailability of the insurance data, small sample size and a short duration of the project. However, these findings do reiterate the importance of screening patients for ACEs and ensuring that they are provided with the required timely education and resources.

## Future Directions/Recommendations

The TIC approach adopted by prenatal care providers in this clinic should continue to include initiating assessment, education, addressing health disparities and most importantly, establishing patient safety and trust (Johnson et al., 2023). Providers should also discuss resilience factors when inquiring about adversity and trauma. Individualized care plans for patients should include evidence-based educational resources, prevention and early intervention programs (Johnson et al., 2023). At this clinic, timely referral to CHWs is an early initiative point for the patients. Thus, ensuring that providers have ACEs and TIC awareness can go a long way in ensuring a timely referral of patients to appropriate resources.

All pregnant patients, even those who are at low risk and without associated health conditions, need to be provided with education about ACEs, toxic stress, and resilience (CDHCS, 2020). Also, there is a need to assess them for protective factors such as a loving, stable support person in their life, and one in their child’s life, a safe neighborhood, and access to fresh, nutritious food. Those patients who are at an intermediate risk with related health conditions or those with high risk with or without associated health conditions need to be provided education about ACEs and their likely role in the development of these conditions.

While universal screening for ACEs in pregnant women is ideal, barriers have been identified. Lack of provider time, training on ACEs, cultural competence, language barriers, patient discomfort in disclosing sensitive personal information, and privacy issues may all interfere with carrying out ACEs screening (Tran et al., 2022). Further research is necessary to identify best practices in these areas. Additionally, assessment of ACEs in pregnant women and pregnancy intention may provide additional information regarding a pregnant patient’s mental health, need for health education, contraception, mental health referrals, social supports, and/or childcare. (Testa et al., 2021). Lastly, the authors recommend the universal implementation of TIC in all healthcare settings (Kopstick et al., 2025; Racine et al. 2020).

## Conclusion

This QI project highlights feasibility of integrating ACEs screening in pregnant women, with the plan to include mental health referrals when warranted. It also shows the statistical and clinical significance of the data for the first time since the initiation of the ACEs screening program at the clinic. It also provides demographic details about the patients who had an ACE score of three or higher. This data also provide an insight into the importance of TIC training for the providers. Lastly, there needs to be a continued conversation about prevention of ACEs, assessment of social determinants of health, building resilience, and encouraging patient participation in the treatment plan. This is facilitated when patients are provided with support services, appropriate referrals with a response assessment and follow up visit scheduled (CDHCS, 2020).

## Figures and Tables

**Fig. 3:**
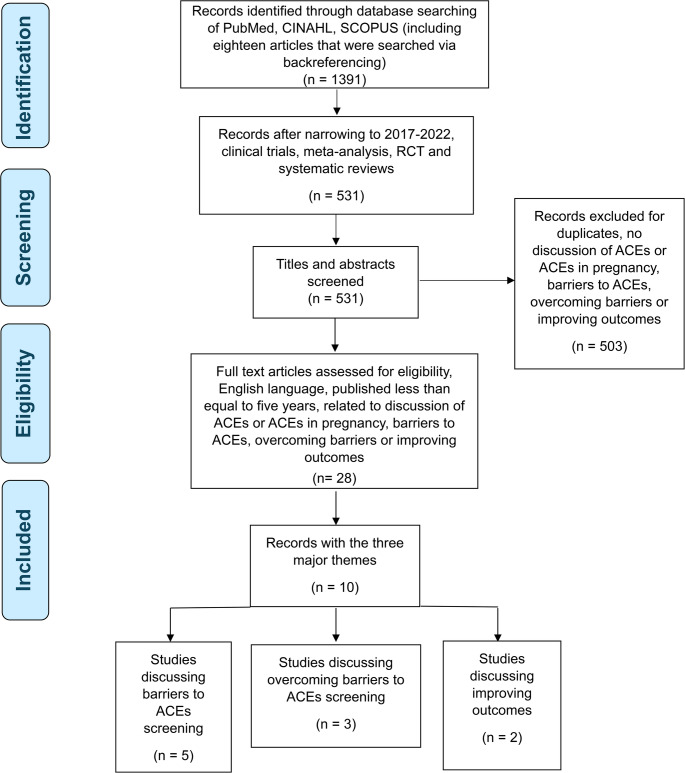
The PRISMA flow diagram of the review of the literature: detailing the databases searched, abstracts screened, and full text articles included

## Data Availability

Not Applicable.
